# Proton-transfer pathways in the mitochondrial *S. cerevisiae* cytochrome *c* oxidase

**DOI:** 10.1038/s41598-019-56648-9

**Published:** 2019-12-27

**Authors:** Markus L. Björck, Jóhanna Vilhjálmsdóttir, Andrew M. Hartley, Brigitte Meunier, Linda Näsvik Öjemyr, Amandine Maréchal, Peter Brzezinski

**Affiliations:** 1 Department of Biochemistry and Biophysics, The Arrhenius Laboratories for Natural Sciences, Stockholm University, SE-106 91 Stockholm, Sweden; 2 Department of Biological Sciences, Birkbeck University of London, Malet Street, London, WC1E 7HX UK; 3 Institute for Integrative Biology of the Cell (12BC), CEA, CNRS, Université Paris-Sud, Université Paris-Saclay, 91198 Gif-sur-Yvette, France; 4 Department of Structural and Molecular Biology, University College London, Gower Street, London, WC1E 6BT UK

**Keywords:** Biophysical chemistry, Oxidoreductases, Bioenergetics

## Abstract

In cytochrome *c* oxidase (Cyt*c*O) reduction of O_2_ to water is linked to uptake of eight protons from the negative side of the membrane: four are substrate protons used to form water and four are pumped across the membrane. In bacterial oxidases, the substrate protons are taken up through the K and the D proton pathways, while the pumped protons are transferred through the D pathway. On the basis of studies with Cyt*c*O isolated from bovine heart mitochondria, it was suggested that in mitochondrial Cyt*c*Os the pumped protons are transferred though a third proton pathway, the H pathway, rather than through the D pathway. Here, we studied these reactions in *S. cerevisiae* Cyt*c*O, which serves as a model of the mammalian counterpart. We analyzed the effect of mutations in the D (Asn99Asp and Ile67Asn) and H pathways (Ser382Ala and Ser458Ala) and investigated the kinetics of electron and proton transfer during the reaction of the reduced Cyt*c*O with O_2_. No effects were observed with the H pathway variants while in the D pathway variants the functional effects were similar to those observed with the *R. sphaeroides* Cyt*c*O. The data indicate that the *S. cerevisiae* Cyt*c*O uses the D pathway for proton uptake and presumably also for proton pumping.

## Introduction

Cytochrome *c* oxidase (Cyt*c*O) is a membrane-bound enzyme that catalyzes reduction of O_2_ to water and uses part of the free energy of this reaction for proton pumping from the negative (*n*) to the positive (*p*) side of the membrane. The electron donor for Cyt*c*O is cytochrome *c* (cyt. *c*), which binds near the electron-entry site, Cu_A_. From Cu_A_ electrons are transferred consecutively to heme *a*, and then to the catalytic site, composed of heme *a*_3_ and Cu_B_. For each O_2_ reduced to water, a total of eight protons are taken up from the *n*-side of the membrane. Four of these protons are used for reduction of O_2_ to H_2_O at the catalytic site (O_2_ + 4 H^+^  + 4 e^−^ − > 2 H_2_O) and the other four protons are taken up from the *n* side and released to the *p* side of the membrane (pumped).

**Figure 1:**
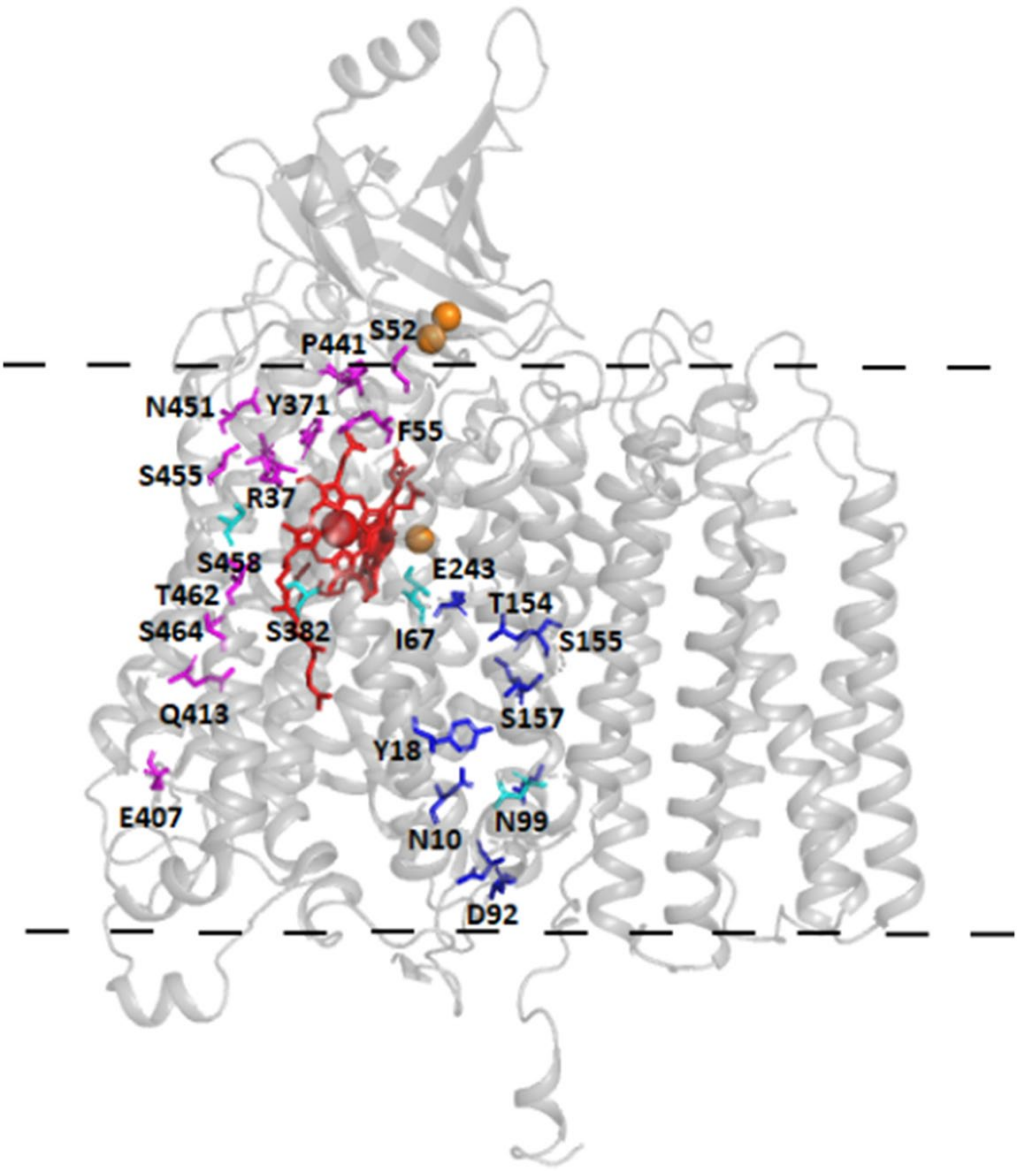
Structural model. The three core subunits of the *S. cerevisiae* Cyt*c*O with the D (blue) and H (magenta) pathway residues marked. Residues that have been substituted in this work are colored in cyan. Copper atoms are colored in orange and hemes in red. The approximate position of the membrane is marked with a dashed line. The structure is based on PDB entry 6HU9^17^.

In the A-type^1,2^ bacterial Cyt*c*Os, protons are taken up through two pathways named the K pathway, after a conserved Lys residue approximately in the middle of the pathway, and the D pathway, named after a conserved Asp residue at the orifice of the pathway (for review, see^3–5^) (Fig. 1). The K pathway is used for uptake of ~2 H^+^ upon reduction of the catalytic site while the D pathway is used for transfer of the remaining 6 protons, i.e. four that are pumped and the remaining two protons used for reduction of O_2_ to H_2_O at the catalytic site. In the B-type oxidases all protons are transferred through a single pathway, which approximately overlaps in space with the K pathway in the A-type oxidases^3,6,7^. Hence, there are alternative pathways for transfer of the pumped protons from the *n*-side in different oxidases.

The mammalian mitochondrial Cyt*c*Os belong to the A-class, i.e. they harbor both the K and D proton pathways. However, structural studies have revealed structural changes in another region of the protein, highlighting a putative third functional pathway called the H pathway^8^ (Fig. 1). The H pathway starts on the *n*-side, near a His residue (His413, bovine Cyt*c*O numbering) and spans the membrane domain close to heme *a* toward the *p*-side, via Asp51 and Ser205 (subunit II), and the Tyr440-Ser441 peptide bond, which has been proposed to control unidirectional proton transfer^9^. It is characterized in its lower part by a water-containing cavity, which was shown to adopt open or closed configuration in different redox and ligand states depending on the interaction of Ser382 with the hydroxyfarnesyl-ethyl group of heme *a*^10^. Pumping measurements were performed on mammalian H pathway variants using a chimeric human/bovine mutagenesis system and the authors concluded that the H pathway, and not the D pathway, was the route taken by pumped protons in mammalian Cyt*c*Os^8,11,12^. Equivalent studies in Cyt*c*O from *R. sphaeroides* confirmed that the H pathway is not involved in proton conduction in bacterial Cyt*c*Os^13^. Mammalian and bacterial Cyt*c*Os have thus been proposed to operate with different proton-pumping mechanisms. An alternative role has also been suggested for the H pathway as a dielectric well that could modulate the effects of buried charge changes on heme *a*^4^.

The bacterial A-type Cyt*c*Os are typically composed of the three core subunits I-III, in some cases having an additional fourth subunit that in e.g. *R. sphaeroides* consists of a single transmembrane helix^14^. The mammalian Cyt*c*Os are bigger with an additional 10–11 accessory subunits^15^. The *S. cerevisiae* mitochondrial Cyt*c*O is an ideal model of the mammalian counterpart^16^. It is composed of the three core subunits as well as 9 accessory subunits^17–20^. An atomic model of *S. cerevisiae* Cyt*c*O determined recently^17^ confirmed its similarity to mammalian Cyt*c*Os, including the core subunits responsible for catalysis and pumping.

In the present study, we prepared structural variants of the *S. cerevisiae* Cyt*c*O in which residues of the proposed D and H pathways were modified. The kinetics of internal electron transfer, were studied in these structural variants. Similar effects were observed with the *S. cerevisiae* Cyt*c*O as with the *R. sphaeroides* Cyt*c*O for the D pathway variants. We did not observe any functional effects of changes in the H pathway of the *S. cerevisiae* Cyt*c*O. Collectively, the data indicate that the mitochondrial *S. cerevisiae* Cyt*c*O displays similar functional characteristics to those seen with bacterial Cyt*c*Os.

## Results

We investigated two D pathway structural variants (Asn99Asp and Ile67Asn) and two H pathway variants (Ser382Ala and Ser458Ala). These structural variants were chosen from a series of modified yeast strains that displayed apparent respiratory growth defects^21^. In earlier studies, reduced minus oxidized difference spectra were recorded of samples of each mutant Cyt*c*O after affinity chromatography purification. These difference spectra of Asn99Asp and Ser382Ala were identical to that of the wild-type Cyt*c*O^21^ whereas those of Ile67Asn and Ser458Ala both displayed a 2–3 nm shift of their alpha-bands to 601 nm^22^ and 606 nm, respectively. A comparison of the numbering of these residues in Cyt*c*Os from *S. cerevisiae*, *R. sphaeroides* and bovine heart mitochondria is shown in Table 1.

**Table 1:** Residues in the D and H pathways.

S. cerevisiae	Bovine	R. sphaeroides
**D pathway**		
D92	D91	D132
N99	N98	N139
I67	I66	M107
E243	E242	E286
**H pathway**		
R37	R38	R52
S52	D51	G92
F55	Y54	W95
Y371	Y371	Y414
S382	S382	S425
E407	D407	E450
Q413	H413	H456
M428	Q428	Q471
I440	Y440	Y483
P441	S441	I484
N451	N451	N494
A454	S454	S497
S455	S455	S498
S458	S458	A501
S464	A464	S507
A461	S461	S504
A230_II_	S205_II_	A261_II_

Equivalent residues in the bovine heart and *R. sphaeroides* Cyt*c*Os are listed.

### Reaction of the reduced CytcO with O_2_

**Figure 2:**
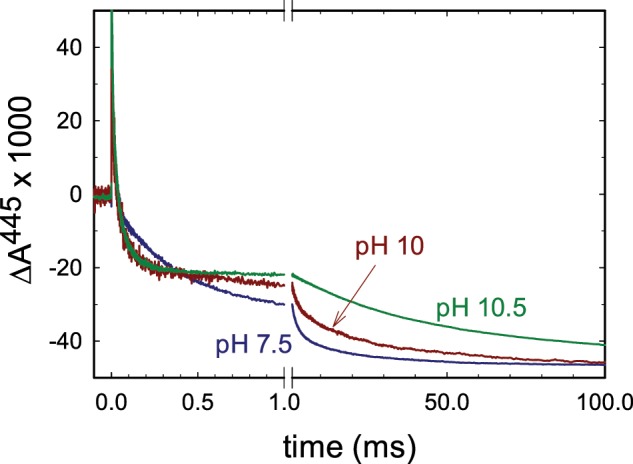
Absorbance changes associated with reaction of the fully reduced Cyt*c*O with O_2_. The sample was illuminated by a laser flash, which results in dissociation of the CO ligand (increase in absorbance at *t* = 0). The decrease in absorbance is associated with binding of O_2_ and formation of the **P**_**R**_ state with a time constant ~40 µs. The slowest decay in absorbance is associated with the **F** → **O** reaction. Representative traces at pH 7.5, 10 and 10.5 are shown. Experimental conditions: mixing ratio of the O_2_-saturated buffer to enzyme was 5:1. The delay time between mixing and the laser flash was 200 ms. The CytcO concentration was ~1.6 µM after mixing. The composition of the CytcO solution was: 150 mM KCl, 10 mM Bis-tris propane pH 7 and 0.035% DDM. The O_2_-containing buffer solution was composed of ~1.2 mM O_2_, 150 mM KCl, 100 mM buffer (MES pH 6, Bis-tris propane pH 7, 7.5, and 9, CHES pH 9.5 and CAPS for pH 10 and 10.5). The traces have been scaled to the same CO-dissociation absorbance change.

To investigate the kinetics of electron transfer in the wild-type and structural variants of the Cyt*c*O, the enzyme was fully reduced (by four electrons) and incubated under an atmosphere of carbon monoxide, which binds at heme *a*_3_, i.e. the ligand blocks access to O_2_. The sample was then rapidly (~2 ms) mixed with an O_2_-saturated solution in a stopped-flow apparatus. Because CO dissociation in the dark is slow (~30 s), the Cyt*c*O stays essentially fully reduced over a time scale of milliseconds. About 200 ms after mixing, the ligand was dissociated by means of a short (~10 ns) laser flash, which initiates the reaction of the rescued Cyt*c*O with O_2_ simultaneously in the entire Cyt*c*O population. Figure 2 shows absorbance changes associated with reaction of the reduced wild-type Cyt*c*O with O_2_ at 445 nm at pH 7.5 and pH 10. The initial increase in absorbance at *t* = 0 is associated with CO dissociation. The decrease in absorbance with a time constant of ~40 µs is associated with electron transfer from heme *a* to the catalytic site forming the peroxy state (**P**_**R**_). This reaction is not associated with any proton uptake from solution and displays a very small pH dependence. In the next step a proton is taken up from solution to form the ferryl state, **F** with a time constant of ~100 µs at pH 7.5. This reaction is mainly seen at 580 nm where absorbance changes are small and it could not be resolved in this study. At 445 nm the reaction is observed as a small lag before the decrease in absorbance with a time constant of ~1 ms at pH 7.5. In this final step of the reaction the **F** state decays to form the oxidized Cyt*c*O (state **O**), which is associated with proton uptake from solution and proton pumping.

**Figure 3:**
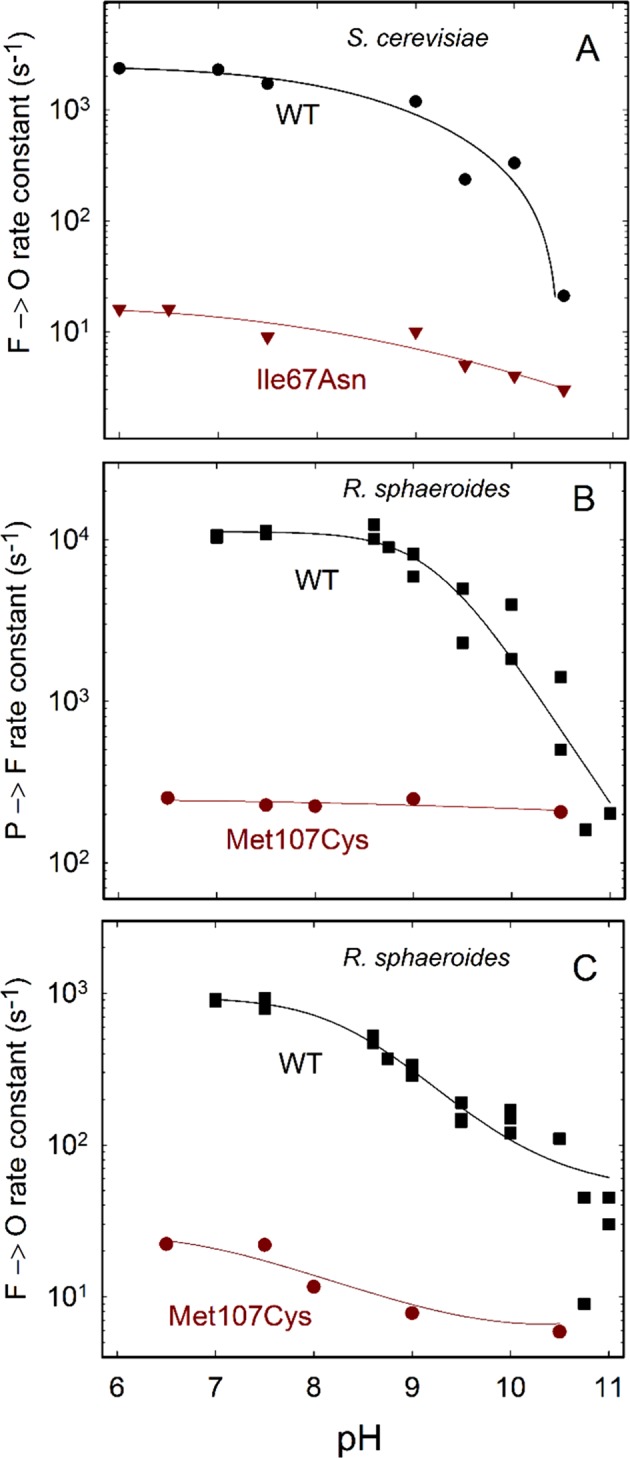
pH dependence of reaction rate constants. (**A**) The rates were determined by fitting an exponential function to traces obtained at 445 nm, after initiation of the reaction of reduced Cyt*c*O with O_2_. Data with the wild-type and I67N Cyt*c*Os. Standard errors ((SD/√*n*; SD, standard deviation, *n*, number of measurements) were typically ~10% of the measured value. Experimental conditions were the same as those in Fig. 2. The different buffers used at different pH values are listed in the Fig. 2 legend. (**B,C**) Data with the *R. sphaeroides* Cyt*c*O. Absorbance changes were measured at 580 nm with the Met107Cys variant and the **P**_**R**_ → **F** (**B**) and **F** → **O** (**C**) rate constants were determined from a fit of a sum of exponential functions (see e.g.^37^). Standard errors were typically ~5% of the measured value (SD of ~20 traces measured with 2 samples). The wild-type data are from^38^. The experiments were carried out in the same way as that in Fig. 2, except that the composition of the Cyt*c*O solution was 10 mM Bis-tris propane pH 7.5, 0.05% DDM and 0.1 mM EDTA. The O_2_-containing buffer solution was composed of ~1.2 mM O_2_, 100 mM buffer (Bis-tris propane pH 6.5, 7.5, 8 and 9 or CAPS for pH 10.5), 0.05% DDM and 0.1 mM EDTA. The solid line is a guide for the eye.

Figure 3A shows the pH dependence of the **F** → **O** reaction. The rate constant for the wild-type enzyme dropped from ~2·10^3^ s^−1^ at low pH to ~20 s^−1^ at pH 10.5. This pH dependence is qualitatively similar to that observed previously with the Cyt*c*O from *R. sphaeroides* or from bovine heart mitochondria^23,24^. A single kinetic component was observed in the pH range 6–9 and at pH 10.5, but at the intermediate pH values the decay was biphasic with approximately equal amplitudes of the two components. For these points an average of the two rates is plotted in Fig. 3A. With the two H pathway variants, Ser382Ala and Ser458Ala, the rates at pH 7 and pH 10 were similar to those observed with the wild-type Cyt*c*O (Table 2).

**Table 2:** **F** → **O** rate constants. Standard errors are shown.

Sample	rate constant (s^−1^) pH 7	rate constant (s^−1^) pH 10
wild type	2200 ± 140 (6)	330 ± 30 (12)
N99D	1200 ± 70 (18)	780 ± 40 (18)
S382A	1200 ± 60 (8)	250 ± 150 (8)
S458A	2500 ± 60 (5)	350 ± 30 (5)

Number of measurements in parentheses. For the S382A mutant Cyt*c*O the error obtained at pH 10 is estimated based on the range of rate constants that could be used to fit an average trace composed of data from eight measurements. In this case, the signal-to-noise ratio did not allow fits of data obtained with the individual samples.

Residue Asn99 is found near Asp92, which is located at the orifice of the D pathway (see Fig. 1). With the Asn99Asp mutant Cyt*c*O, we observed **F** → **O** rate constants of 1200 ± 100 s^−1^ and 780 ± 100 s^−1^ at pH 7 and pH 10 (10 measurements on each of two samples), respectively, i.e. the rate constant was larger with the Asn99Asp than with the wild-type Cyt*c*O at pH 10, consistent with earlier observations made with the *R. sphaeroides* Cyt*c*O^25^.

Residue Ile67 is located at a distance of ~3 Å from Glu243, a key D pathway residue, at the end of the pathway (see Fig. 1). With the Ile67Asn, the **F** → **O** rate was about a factor of 100 slower than that seen with the wild-type Cyt*c*O, which is consistent with proton uptake through the D pathway in this reactions step. To test the effect of this structural alteration in a bacterial Cyt*c*O, we introduced a mutation at the equivalent residue, Met107 in the *R. sphaeroides* Cyt*c*O, which is also located ~3 Å from Glu286 (equivalent of Glu243 in *S. cerevisiae*). In both *S. cerevisiae* (Ile67Asn) and *R. sphaeroides* (Met107Cys) mutation at this site resulted in lower Cyt*c*O activity of ~10%^22^ and ~6%, respectively (see “Materials and Methods”). With the *R. sphaeroides* mutant Cyt*c*O we obtained sufficient amount of Cyt*c*O material to be able to perform measurements also at 580 nm where the **P**_**R**_ → **F** reaction step is clearly observed as an increase in absorbance in the time range (Fig. 3B). As seen in the figure, the **P**_**R**_ → **F** reaction rate constant was a factor of ~50 slower than that observed with the wild-type Cyt*c*O. Because the absorbance changes associated with the **P**_**R**_ → **F** reaction reflect proton uptake through the D pathway^26,27^, the data indicate that in the *R. sphaeroides* Cyt*c*O, the amino-acid residue replacement slows proton uptake through this pathway, presumably due to an interaction with Glu286. Note that at high pH, i.e. when proton uptake from solution becomes rate limiting for the **P**_**R**_ → **F** reaction, similar rate constants were observed with the wild-type and mutant Cyt*c*Os. Similar to the results obtained with the *S. cerevisiae* Cyt*c*O, the replacement of Met107 by a Cys in *R. sphaeroides* Cyt*c*O resulted in slowing the **F** → **O** reaction rate constant by a factor of 50 to ~20 s^−1^ at pH 7 (Fig. 3C).

## Discussion

Results from earlier studies with bacterial Cyt*c*Os showed dramatic effects of mutations in the D pathway on the rate of proton transfer and/or the proton-pumping stoichiometry^28^. In the *R. sphaeroides* Asn139Asp variant (Asn99Asp in *S. cerevisiae*) proton pumping was uncoupled from O_2_ reduction while the activity of the Cyt*c*O was slightly increased^29^. The maximum rate of the **F** → **O** reaction at pH < 7 was unaffected by the mutation, but the pH dependence of the reaction rate was altered resulting in an increase in the p*K*_a_ from 9.4 to ~11^25^. Mutation of Asn207 to Asp (Asn164Asp in the *S. cerevisiae* Cyt*c*O), near Asn139, also resulted in uncoupling of proton pumping and an altered pH dependence of the **F** → **O** reaction^25^. No effects on proton pumping were observed with the equivalent mutants in the mammalian Cyt*c*O, which prompted the authors to conclude that the function of the D pathway is not conserved^8,11^ and that the H pathway is involved in proton pumping^11^.

The results from the present study show that the **F** → **O** reaction rate at pH 7 was similar with the Asn99Asp Cyt*c*O as with the wild-type Cyt*c*O (Table 2). As already noted above with the *R. sphaeroides* Cyt*c*O, the characteristic feature of this variant is a shift in the pH dependence of the **F** → **O** rate resulting in an elevated p*K*_a_ value. While this rate constant with the wild-type *R. sphaeroides* Cyt*c*O decreased by a factor of five from ~1000 s^−1^ to ~200 s^−1^ upon increasing the pH from 6 to 10, in Asp139Asn variant it decreased from 1200 s^−1^ to 900 s^−1^, i.e. by a factor of ~1.3^25^.

With the *S. cerevisiae* wild-type Cyt*c*O, the **F** → **O** rate constant decreased from ~2200 s^−1^ at pH 7 to ~330 s^−1^ at pH 10, i.e. by a factor of ~7, while with the Asn99Asp Cyt*c*O the rate decreased from 1200 s^−1^ at pH 7 to ~780 s^−1^ at pH 10, i.e. by a factor of ~1.6 (Table 2). Hence, we observed a similar behaviour with the *S. cerevisiae* enzyme as with the *R. sphaeroides* Cyt*c*O.

The current study shows that structural alterations close to the D pathway, at the site of residue Ile67 in *S. cerevisiae* or Met107 in *R. sphaeroides* Cyt*c*O, result in a dramatically slower **F** → **O** reaction rate (by factors of ~100 and ~50, respectively). This residue is located at hydrogen-bonding distance from the Glu243 (or Glu286 in the *R. sphaeroides* Cyt*c*O). Results from earlier studies have shown that replacement of Glu286 by other residues results in a dramatic decrease or impaired proton transfer through the D pathway^30^. Hence, this segment of the D pathway is particularly sensitive to structural alterations. Because with the *R. sphaeroides* Cyt*c*O we observed a ~50-fold slower **P**_**R**_ → **F** reaction rate with the Met107Cys variant (Fig. 3), the effect of the mutation is a dramatically slowed proton transfer through the D pathway. Hence, we conclude that also with the *S. cerevisiae* Cyt*c*O the slowed **F** → **O** reaction rate is caused by slowed proton transfer through the D pathway. This interpretation is also consistent with earlier FTIR studies which showed that the low activity of the Ile67Asn variant was linked to perturbations of Glu243 and impairment of its proposed role in proton coupled electron transfer^22,31^.

The observations discussed above are consistent with data obtained from studies of the Glu243Asp variant^32^, and, collectively, they indicate that the **F** → **O** reaction is associated with proton transfer through the D pathway also in the *S. cerevisiae* Cyt*c*O. We did not observe any effects on the **F** → **O** rate constants at pH 7 or pH 10, measured with the H pathway variants (see Table 2). Because proton pumping in Cyt*c*O is driven by electron transfer to O_2_ (and the associated uptake of substrate protons for water formation), if proton pumping would be impaired in the H-pathway mutants, we would expect to observe an effect on the O_2_-reduction reaction. Because the oxidation kinetics was unperturbed, we conclude that the functionality of the mutants investigated here is unperturbed.

Data from earlier studies showed that the Ser458 to Ala replacement resulted in a decreased respiratory growth^21^. However, this observation does not imply that proton-pumping would be impaired in the Ser458Ala variant. For example, the mutation could instead yield a fraction CytcO that is not assembled correctly, resulting in slowed overall respiration. In the present study we studied purified Cyt*c*O and the observed signals originate from Cyt*c*O that bind CO to an intact catalytic site. Results from a study with the *R. sphaeroides* Cyt*c*O in which Ser425, the equivalent of the *S. cerevisiae* Cyt*c*O Ser382 residue, was modified, showed that this residue is not important for proton conduction, but only results in a small increase in the midpoint potential of heme *a*^33^.

The data discussed above indicate that the mitochondrial Cyt*c*O from *S. cerevisiae* uses the D pathway for proton transfer from solution to the catalytic site in the **F** → **O** reaction step. Even though here we studied electron transfer to O_2_ at the catalytic site, this electron transfer is linked to proton pumping. Thus, as indicated above, any effect on proton pumping is expected to result in an effect on the intramolecular electron transfer. Mutations of residues in the D pathway displayed qualitatively the same functional characteristics in the *S. cerevisiae* as with the *R. sphaeroides* Cyt*c*O and the **F** → **O** rate was insensitive to changes in the proposed H pathway. Hence, the data suggest that there are no major functional differences between the mitochondrial *S. cerevisiae* and the bacterial *R. sphaeroides* Cyt*c*Os.

## Methods

### Preparation of mutants

The construction of the modified yeast strains is described in^21^.

To prepare the Met107Cys Cyt*c*O variant in *R. sphaeroides* a Quik-Change II site-directed mutagenesis kit (Agilent technologies) was used. The resulting amino-acid replacement was verified by sequencing. The PJS3-SH plasmid was used as a template plasmid for making mutations while pRK415-1 plasmid^34^ was used for expression^35^.

### Preparation of the ***S. cerevisiae*** Cyt*c*O

Yeast cells were grown aerobically in YPGal medium at 28 °C and harvested in late log phase as described in^21^. Mitochondrial membranes were prepared and the *S. cerevisiae* Cyt*c*O was purified as described by Meunier *et al*.^21^ with some modifications as outlined here. During solubilization the membranes were diluted to 2 mg/ml in buffer (50 mM KPi, 100 mM KCl and 1.5% *n*-dodecyl β -D-maltoside (DDM (w/v)) and solubilized for 1 hour. To remove cyt. *c* the supernatant from the centrifugation step after solubilization was run over a column loaded with a cation-exchanger (Bio-Rex 70, Bio-Rad) equilibrated with 50 mM KPi, 100 mM KCl and 0.035% DDM. After addition of 5 mM imidazole to the flow-through from the ion-exchanger it was loaded on a column filled with 25 ml Ni-resin (NI Sepharose 6 Fast Flow from GE healthcare), pre-equilibrated with (20 mM KPi, 150 mM KCl and 0.035% DDM). The column was washed with four column volumes of wash buffer (20 mM KPi, 150 mM KCl, 10 mM imidazole and 0.035% DDM). This was followed in time by a two-step elution of four column volumes at each imidazole concentration (20 mM KPi, 150 mM KCl, 40 or 100 mM imidazole and 0.035% DDM). The eluted fractions were pooled and concentrated in a 100 kDa cut-off filter (Merck Millipore). Buffer exchange (elution buffer without imidazole) was performed to lower the imidazole content to sub-µM concentrations.

### Preparation of the *R. sphaeroides* Cyt*c*O

Expression of the *R. sphaeroides* Cyt*c*O was achieved by growing bacteria aerobically (Sistrom medium) in the dark at 30 °C. After harvesting the cells (at OD_550_ ≅ 1.5) they were re-suspended (50 mM Tris-buffer, pH 8.0) at 4 °C in the presence of DNase I (0.05 mg/ml final concentration) and passed twice through a continuous-flow cell disruptor (Constant Systems LTD) operating at 170 MPa. The inner membrane fraction was collected by ultracentrifugation (138 000 g for 90 minutes at 4 °C) and 1.5% DDM was added to solubilize the membrane fraction. The histidine-tagged Cyt*c*O was purified using Ni^2+^-NTA affinity chromatography, essentially as described in^35,36^.

### Activity

A Clark-type O_2_ electrode (Hansatech instruments) was used to measure the oxygen reduction rate of the Met107Cys Cyt*c*O. A buffer composed of 50 mM KPi (pH 6.7) and 0.1% DDM was added to the reaction chamber and supplemented with 6 mM ascorbate, 670 µM *N*,*N*,*N*,*N*-tetramethyl-*p*-phenylenediamine (TMPD) and 32 µM cyt. *c*. A Cyt*c*O solution (in 100 mM Hepes, pH 7.5 and 0.05% DDM) was added (final concentration of 7 nM) to the reaction chamber and the oxygen-consumption rate during Cyt*c*O turnover was monitored (a background oxygen consumption before addition of the Cyt*c*O was subtracted). The steady state activity of the Met107Cyt CytcO variant was ~6% of that of the wild-type Cyt*c*O, which was ~570 e^−^/s/Cyt*c*O.

### Flow-flash measurements

The sample was prepared by exchanging the Cyt*c*O buffer to 10 mM (wild-type, Ile67Asn and Met107Cys variants) or 3 mM (Asn99Asp, Ser382Ala and Ser458Ala)) Bis-tris propane pH 7, 150 mM KCl and 0.035%DDM. The Cyt*c*O concentration after buffer exchange was 3-10 µM depending on mutant (see figure legends). The sample was transferred to a Thunberg cuvette and air was exchanged for nitrogen. The sample was then reduced with 4 mM ascorbate and 1 µM PMS (electron mediator) (*S. cerevisiae* Cyt*c*O) or 2 mM ascorbate and 1 µM ruthenium hexachloride (electron mediator) (*R. sphaeroides* Cyt*c*O). Nitrogen was then exchanged for CO. The absorbance spectra were recorded in the range 400–700 nm at each step to ensure complete reduction and ligation with CO.

The sample was loaded into one syringe of a modified stopped-flow apparatus (Applied Photophysics). The other syringe was loaded with an oxygen-saturated buffer. The buffer composition for measurements with the *S. cerevisiae* Cyt*c*O: 100 mM (for 1:5 mixing of Cyt*c*O:O_2_ solutions) or 200 mM buffer (for 1:1 mixing) MES for pH 6 and 6.5; Bis-tris propane pH 7, 7.5 and 9; CHES for pH 9.5 and CAPS for pH 10 and 10.5. In addition, the buffer was supplemented with 150 mM KCl and 0.035% DDM. For measurements with *R. sphaeroides* Cyt*c*O: 100 mM Bis-tris propane buffer for pH 6.5, 7.5, 8 and 9; CAPS for pH 10.5 with 0.05% DDM and 0.1 mM EDTA. The CO ligand was dissociated at 0.2 s after mixing, which initiated the reaction. The reaction was monitored by following in time absorbance at specific wavelengths (see figure legends). An amplifier (C11184, Hamamatsu) was used to amplify the signal before recording using a digital oscilloscope.
